# Housing status, mental health, and help-seeking among American Indian and Alaska Native Veterans in the Midwest

**DOI:** 10.3389/fpubh.2025.1613224

**Published:** 2025-06-18

**Authors:** Emre Umucu, Beatrice Lee, Chi Chang, Jack Tsai

**Affiliations:** ^1^Department of Public Health Sciences, College of Health Sciences, The University of Texas at El Paso, El Paso, TX, United States; ^2^CHS REACHED, College of Health Sciences, The University of Texas at El Paso, El Paso, TX, United States; ^3^Audie L. Murphy Memorial Veterans’ Hospital, South Texas Veterans Health Care System, San Antonio, TX, United States; ^4^Department of Rehabilitation Sciences, College of Health Sciences, The University of Texas at El Paso, El Paso, TX, United States; ^5^Office of Medical Education Research and Development, College of Medicine, Michigan State University, East Lansing, MI, United States; ^6^US Department of Veterans Affairs, National Center on Homelessness Among Veterans, Washington, DC, United States; ^7^School of Public Health, University of Texas Health Science Center at Houston, Houston, TX, United States; ^8^Department of Psychiatry, Yale University School of Medicine, New Haven, CT, United States

**Keywords:** American Indian and Alaska Native, veterans, mental health, service utilization, homelessness, rural and frontier

## Abstract

**Objective:**

This study aims to examine mental health and help-seeking behaviors among homeless and unstably housed (HUH) and stably housed (SH) American Indian and Alaska Native (AIAN) and non-AIAN veterans in the Midwest.

**Methods:**

The study cohort consisted of veterans in the Veterans Affairs (VA) service catchment area called the Veterans Integrated Service Network (VISN) 23. Data from the Homeless Operations Management and Evaluation System (HOMES) were analyzed with descriptive statistics and the Mantel–Haenszel odds ratio with Wald confidence intervals, and the Breslow-Day test.

**Results:**

Of the 7,260 veterans in the study, 5,771 (80/9%) were HUH. Among non-AIAN participants, 5,453 (80.8%) experienced HUH, compared to 318 (84.6%) of AIAN veterans. The Breslow-Day test revealed significant differences in the association between housing status and certain service needs between AIAN and non-AIAN veterans.

**Conclusion:**

This study highlights notable differences in housing status, mental health conditions, and service needs between AIAN and non-AIAN veterans in the Midwest.

## Introduction

American Indian and Alaska Native (AIAN) veterans have served in the military at a higher rate compared to the national average (1, 2). The Department of Veterans Affairs (VA) revealed that the AIAN veteran cohort served in the Pre-9/11 period of service at a higher percentage than non-AIAN veterans (17.7% vs. 14.0%, respectively) (1). Moreover, AIAN veterans are overrepresented in combat roles, increasing their exposure to combat-related challenges. This heightened exposure can lead to poorer post-military health outcomes and adjustment difficulties compared to veterans of other races (3–6). These experiences may contribute to disproportionately negative outcomes for AIAN veterans, including increased health disparities and higher rates of homelessness and unstable housing (HUH) (7, 8).

### Homelessness and housing instability among AIAN veterans

Homelessness poses a significant public health concern, that may exacerbate existing health disparities within the AIAN veteran population (7, 9–11). A national study of veterans within the VA healthcare system revealed that AIAN veterans face a greater risk of HUH compared to white veterans. Furthermore, AIAN women veterans demonstrated a significantly higher likelihood of experiencing HUH than Asian/Pacific Islander and white male veterans (56% compared to 15.6 and 2.1%, respectively) (7). The elevated rates of HUH among AIAN veterans may be partly attributed to existing disparities within the general AIAN population, such as inadequate healthcare funding, disproportionate poverty, economic adversity, poor social conditions, and discrimination in healthcare delivery (12).

There are certain factors that contribute to HUH among AIAN veterans. One of the most important factors is that AIAN veterans often experience lower levels of education and higher unemployment rates compared to other racial/ethnic groups (2, 13, 14), factors known to significantly increase the risk of HUH among veterans (15, 16). Furthermore, there issues are not unique to AIAN veterans, and in fact, a body of literature has documented the negative health and social outcomes of AIAN youth, adults, and older adult populations; which are rooted in a history of displacement dating back to the U.S. settlers and decades of government policies that negatively affected generations of AIAN individuals and families (16–19).

### Mental health conditions among AIAN veterans

Research revealed that AIAN civilians and veterans are at greater risk for mental health conditions (2, 20–22). Beals et al. (20) explored the prevalence of posttraumatic stress disorder (PTSD) among AIAN Vietnam veterans and found that AIAN veterans were at higher risk of PTSD compared to White samples. They also reported that AIAN Vietnam veterans had higher rates of lifetime and current PTSD diagnoses compared to white veterans. Furthermore, VA published a report on veteran health equity (23) and reported that PTSD and depression were diagnosed more frequently in AI/AN Veterans compared to white Veterans (20.7% vs. 12.1% respectively). Smith et al. (24) examined race/ethnic differences in the prevalence and co-occurrence of substance use disorders and reported that over a 12-month period, AIAN typically had the highest rates of mood, anxiety, and substance use disorders. Similarly, Koo et al. (25) reported that PTSD and alcohol use were highest among AIAN men and women compared to other race and ethnicity groups.

#### Access to care among AIAN veterans

While AIAN veterans face higher levels of psychiatric conditions compared to veterans of other races, they may also encounter more significant challenges in accessing appropriate healthcare services (26). These challenges are multifaceted and include cultural issues, transportation difficulties, navigating complex healthcare systems, low health literacy, and residing in rural and frontier (i.e., any territory characterized by some combination of low population size and high geographic remoteness) areas (4, 26–28). Additionally, AIAN veterans are less likely to have health insurance compared to veterans of other races (1). A study examining healthcare disparities for AIAN veterans in the US reported that AIAN veterans had 1.9 times higher odds of being uninsured compared to white veterans (29).

AIAN veterans have reported facing challenges in accessing services from the Indian Health Services (IHS) due to the underfunding of many IHS programs (29). This persistent underfunding has resulted in healthcare rationing, impacting even AIAN individuals who are eligible for these services (29). It is also crucial to remember that AIAN veterans are approximately four times more likely than non-AIAN veterans to reside in rural settings, further exacerbating barriers to accessing healthcare services (4). The limited access to culturally competent and affordable healthcare services can lead to untreated physical and mental health conditions, increasing the risk of HUH and other adverse outcomes within this population (30).

#### Help-seeking behaviors among AIAN veterans

In addition to environmental and structural challenges that hinder AIAN veterans’ access to healthcare, AIAN veterans may have other concerns as well, such as cultural mistrust of mental healthcare systems, stemming from historical experiences of trauma and discrimination (1), and cultural-based views of help-seeking for psychiatric disorders as stigmatizing (31). This stigma can lead to feelings of shame and apprehension when seeking mental health services (31). A study exploring perceived barriers to VA mental health care among upper Midwest American Indian veterans revealed that family and community support for mental health care was often lacking due to stigma and shame (32). Additionally, VA data showed that a smaller proportion of AIAN veterans (41.6%) utilized at least one VA benefit or service compared to veterans of other races (52.7%) (1). Although this does not directly suggest that AIAN veterans may be less inclined to seek assistance compared to their peers from other racial groups, it highlights the complex patterns of service use among AIAN veterans.

Given that AIAN veterans face challenges in accessing mental health care services due to environmental and structural barriers, such as being located in remote areas, it is crucial to understand their mental health help-seeking behaviors. In addition, historical trauma in AIAN is associated with poor mental health (33, 34). Overall, AIAN veterans, especially those who are HUH, may be more vulnerable to significant health disparities that need to be addressed by researchers. Therefore, understanding mental health and help-seeking behaviors among HUH AIAN veterans will enable the development of targeted interventions aimed at reducing health disparities among AIAN veterans.

### Purpose

The concentration of HUH research on the Eastern and Western coasts of the U.S. has resulted in a significant knowledge gap regarding Midwest regions, where the issue remains underexplored, despite the significant population of AIAN veterans residing there. The Midwest may have unique regional challenges associated with socioeconomic conditions, access to healthcare resources, housing resources, cultural stigma surrounding mental health, and varying levels of community support. Investigating mental health and help-seeking behaviors among HUH AIAN veterans in the Midwest is crucial, as it can reveal unique regional challenges and disparities not addressed in coastal studies. Understanding these specific factors could provide valuable insights for developing targeted interventions and policies that address the distinct needs of AIAN veterans in the Midwest, ultimately contributing to more effective solutions for homelessness nationwide. Comparing AIAN and non-AIAN veterans can help us understand unique mental health disparities in mental health and housing outcomes, service usage, and barriers to care. This will also provide us with insights that will inform policy and interventions to address the needs of both AIAN and non-AIAN veterans.

To address the existing gap in the literature on mental health and help-seeking behaviors among HUH AIAN veterans, we conducted a study of AIAN and non-AIAN veterans residing in several Midwestern states. Our study aimed to: (1) ascertain the proportion of mental health conditions among veterans, stratified by housing status and AIAN status; (2) examine the interaction effect of AIAN status on the relationship between psychiatric disorders and housing status among veterans; (3) assess service needs and willingness to seek help among veterans; (4) explore the interaction effect of AIAN status on the relationship between housing status and service needs, and (5) explore the interaction effect of AIAN status on the relationship between housing status and the willingness to seek help among veterans.

## Methods

### Procedure

The study cohort consisted of veterans in the VA service catchment area called the Veterans Integrated Service Network (VISN) 23, which includes the states of North and South Dakota, Iowa, Minnesota, and Nebraska. The study cohort included stably housed (SH) and HUH veterans in VISN 23 who completed an assessment/intake form for a VA homeless program from 2018 to 2022. Data on entries and exits from the VA homeless programs were extracted from the Homeless Operations Management and Evaluation System (HOMES) in 2022, except the data on Supportive Services for Veteran Families (SSVF) utilization, which was extracted from the Homeless Management Information System (HMIS). Using a veteran’s first HOMES assessment as the index date, we used VA’s Corporate Data Warehouse (CDW) to extract all diagnoses recorded in the veteran’s inpatient and outpatient records in the year prior to this index date. These analyses were conducted as part of a quality improvement project at the request of and funded by VISN 23 in coordination with the National Center on Homelessness Among Veterans. This work was therefore not considered human subjects research by the VA institutional review board.

HOMES is a centralized data system used by the VA as part of its medical record system to capture provider workload and service use of veterans who use VA homeless programs (35). HOMES was developed to monitor homeless Veterans as they move through the VA’s system of care, beginning with assessments that generate referrals to appropriate services and continuing through their transitions into and out of VA homeless programs (35).

### Measures

#### Sociodemographic characteristics

Sociodemographic characteristics, retrieved from HOMES assessment data, included age in years; gender (male, female, and transgender); and race/ethnicity (non-Hispanic White, non-Hispanic Black, American Indian/Alaska Native, Hispanic, and Other/Mixed), and education.

#### Military characteristics

Military characteristics included service years; military branch (Air Force, Army, Coastal Guard, Marines, Navy); and combat exposure status (yes/no) and were based on HOMES data.

#### Socioeconomic characteristics

Socioeconomic characteristics included years of education; any work for pay in the past 30 days; percent service-connected disability rating (0%, 10–40%, 50–100%); and debt status (yes/no) were based on VA CDW data.

#### Risky behavior characteristics

Risky behavior characteristics encompass indicators such as any previous jail or prison experience (yes/no), drug abuse (yes/no), alcohol abuse (yes/no), engagement in risky behavior (yes/no), and suicidal ideation (yes/no). The risky behaviors section consists of single items representing five behaviors, which we categorized collectively under risky behavior characteristics. Each item was scored dichotomously as 0 (no) or 1 (yes). These data were retrieved from HOMES data.

#### Geographic characteristics

Geographic characteristics included rurality status (rural, urban) and frontier and remote score (FAR). Rurality status was determined using the Rural–Urban Commuting Area (RUCA) Classification System, developed by the University of Washington in collaboration with state and federal agencies (36). The RUCA system includes two levels: whole numbers (1–10) that represent different types of areas—metropolitan, micropolitan, small town, and rural—based on the size and direction of primary commuting flows. Frontier and remote scores were categorized using the U.S. Department of Agriculture’s 2010 FAR Codes Data files (please see the link [https://www.ers.usda.gov/data-products/rural-urban-commuting-area-codes/] for details) (37). FAR scores range from 0 to 4, where 0 indicates no frontier characteristics, and 4 signifies highly frontier conditions. To classify participants, zip codes were used to match their geographic location with the RUCA and FAR classifications. Based on this matching, participants were categorized into rural versus urban areas using RUCA and into frontier versus non-frontier areas based on their FAR score.

#### Clinical and health characteristics

Clinical and health characteristics included physical health status in the past 30 days (0 = poor to 4 = excellent); chronic pain status (yes/no); severe mental illness status (yes/no); and number of times in the emergency room in the past 6 months. We created indicators for physical health conditions using the Elixhauser algorithm for the International Classification of Diseases (38), version 10 (ICD-10); we also created indicators for psychiatric disorders not included in the Elixhauser algorithm. All physical and psychiatric disorders were dichotomous (yes/no).

#### Homelessness and unstably housed

Conceptually, Tsai et al. (39) defined housing instability “*as the state of living in housing but currently being at-risk of losing that housing”* and homelessness as *“the state of living in a place not meant for human habitation (e.g., outdoors, vehicles), in emergency shelter, in transitional housing, or exiting an institution (hospital, jail) with no permanent housing arrangement*.” Using an operational definition developed by the VA National Center on Homelessness Among Veterans (11, 39), we identified veterans as homeless or unstably housed if they had an ICD-10 code Z59.0, participated in any VA homeless program, or screened positive for homelessness or risk of homelessness on the VA homeless risk screening clinical reminder. Veterans who screened negative for both homelessness and the risk of homelessness were presumed to be stably housed.

#### Service needs and willingness to seek help

Participants’ service needs for psychiatric, substance-use, or medical treatment, as well as assistance with family problems, were assessed using dichotomous items (no = 0 vs. yes = 1). This section of HOMES data had a total of five items measuring participants’ service needs for psychiatric, substance-use, medical treatment, as well as assistance with case management and family problems. For instance, the need for psychiatric treatment was evaluated with the question, “Does this Veteran need psychiatric treatment at this time?” Participants’ willingness to seek these services was also assessed through similar dichotomous questions (no = 0 vs. yes = 1). This section of HOMES data had a total of five items to measure participants’ willingness to seek services reported above. For instance, the need for psychiatric treatment was evaluated with the question, “Is the Veteran interested and willing to participate in psychiatric treatment?” It is important to note that these assessments were made by VA providers based on clinical judgment and were retrieved from HOMES.

### Data analysis

First, descriptive statistics of the total sample were calculated, including means, standard deviations, percentages for study variables, and effect sizes of *d* and *ω*. Second, to compare the prevalence of psychiatric disorders based on AIAN status, we employed the Mantel–Haenszel odds ratio with Wald confidence intervals. In this analysis, AIAN status was used as a grouping variable to compare the prevalence of psychiatric disorders between AIAN and non-AIAN veterans. Third, to determine whether AIAN status also acted as a confounding variable, we conducted preliminary analyses to assess its potential influence on the association between psychiatric disorders and other variables, such as housing status. We also calculated the Breslow-Day statistics to determine whether the association between psychiatric disorders and housing status was consistent for AIAN and non-AIAN veterans. We calculated effect sizes for each association to report the magnitude of the association. Fourth, we calculated the prevalence of help-seeking among veterans with psychiatric disorders. Finally, we examined AIAN status as an interaction variable for the relationship between service needs and willingness to seek help among veterans by calculating Mantel–Haenszel odds ratio with Wald confidence intervals and Breslow-Day test. “The Mantel–Haenszel adjusted measures of association are valid when the measures of association across the different strata are similar (homogenous), that is, when the test of homogeneity of the odds (risk) ratios is not statistically significant” (44, p. 10). The statistical significance level was set at *α* value of 0.05, and odds ratios were reported as well. All statistical procedures were performed using R Studio (40).

## Results

### Descriptive statistics

Of the 7,260 veterans in the study, 5,771 (80/9%) were HUH. Among non-AIAN participants, 5,453 (80.8%) experienced HUH, compared to 318 (84.6%) of AIAN veterans. The mean age was 51.98 years (*SD* = 13.72) for non-AIAN veterans and 49.27 years (*SD* = 14.14) for AIAN veterans. The majority of participants were male, comprising 91.45% of non-AIAN veterans and 84.33% of AIAN veterans. Among non-AIAN veterans, most were non-Hispanic White (*M* = 5,029; 73.1%), followed by non-Hispanic Black (*M* = 1,151, 16.7%), Other/Mixed (*M* = 359; 5.3%), and Hispanic (*M* = 338; 4.9%). The mean years of total education was 13.29 years (*SD* = 1.90) for non-AIAN veterans and 13.08 years (*SD* = 1.88) for AIAN veterans. Regarding marital status, 15.12% of non-AIAN veterans and 17.49% of AIAN veterans reported being married or partnered (see Table 1).

**Table 1:** Descriptive statistics of American Indian and Alaska Native (AIAN) and non-AIAN veterans in Veterans Integrated Service Network (VISN) 23.

Characteristics	Non-AIAN (*N* = 6,877)		AIAN (*N* = 383)		Effect size
	*N* (%)	Mean (SD)	*N* (%)	Mean (SD)	
Sociodemographic					
Age		51.98 (13.72)		49.27 (14.14)	*d* = 0.19^*^
Gender					*ω* = 0.06^*^
Male	6,289 (91.45%)		323 (84.33%)		
Female	563 (8.19%)		60 (15.67%)		
Transgender	25 (0.36%)		0 (0.00%)		
Married/partnered	1,038 (15.12%)		67 (17.49%)		*ω* = 0.01^**^
Military					
Service years		4.95 (4.60)		4.24 (3.16)	*d* = 0.15^*^
Branch					*ω* = 0.07^*^
Air Force	764 (11.28%)		20 (5.29%)		
Army	3,608 (53.26%)		242 (64.02%)		
Coast guard	38 (0.56%)		0 (0.00%)		
Marines	1,019 (15.04%)		67 (17.72%)		
Navy	1,345 (19.86%)		49 (12.96%)		
Exposed to combat	2,005 (29.16%)		134 (34.99%)		*ω* = 0.03^*^
Financial					
Education (years)		13.29 (1.90)		13.08 (1.88)	*d* = 0.11^*^
Employment (past 30 Days)		3.45 (7.70)		3.56 (7.51)	*d* = 0.01^**^
Disability (1–100%)	3,869 (56.30%)		211 (55.09%)		*ω* = 0.01^**^
Debt status (Yes)	4,062 (64.20%)		196 (54.29%)		*ω* = 0.05^*^
Risky behavior					
Experienced jail	4,836 (74.54%)		306 (82.04%)		*ω* = 0.04^*^
Drug abuse (Yes)	1,319 (19.18%)		74 (19.32%)		*ω* = 0.001^**^
Alcohol abuse (Yes)	1,734 (25.21%)		128 (33.42%)		*ω* = 0.04^*^
Risky behavior (Yes)	586 (9.31%)		34 (9.12%)		*ω* = 0.001^**^
Suicidal ideation (Yes)	689 (10.02%)		29 (7.57%)		*ω* = 0.001^**^
Health					
Health status		2.5 (1.03)		2.47 (0.94)	*d* = 0.04^**^
Chronic pain	3,804 (45.58%)		171 (44.76%)		*ω* = 0.001^**^
Severe mental illness	762 (11.08%)		29 (7.57%)		*ω* = 0.03^*^
Any ER (Past 6 Months)	3,311 (48.14%)		191 (49.66%)		*ω* = 0.001^**^
Need any help		2.65 (1.22)		2.48 (1.23)	*d* = 0.13^*^
Geographic locations					
Rural	1,336 (19.80%)		152 (40%)		*ω* = 0.11^*^
Urban	5,413 (80.20%)		228 (60%)		
Frontier and remote score (0–4)		0.15 (0.58)		1.19 (1.67)	*d* = 0.19^*^
Housing status					
Homeless or unstably housed	5,453 (80.8%)		318 (84.6%)		
Stably housed	1,298 (19.2%)		58 (15.4%)		

ER, Emergency room; HUH, Homelessness and Unstable Housing; ω, Cohen’s ω effect size; *d*, Cohen’s d effect size. ^*^p < 0.05, ^**^p = n.s.

The mean years of military service were 4.95 (*SD* = 4.60) for non-AIAN veterans and 4.24 (*SD* = 3.16) for AIAN veterans. A greater proportion of AIAN veterans reported serving in the Army (64.02%) compared to non-AIAN veterans (53.26%). Additionally, a higher proportion of AIAN veterans were exposed to combat (34.99%) compared to non-AIAN veterans (29.16%). A similar proportion of veterans reported disability, with 55.09% among AIAN veterans and 56.30% among non-AIAN veterans. Finally, more non-AIAN veterans reported debt status (64.20%) compared to AIAN veterans (54.29%).

For military characteristics, combat exposure is notably higher among AIAN veterans than among non-AIAN veterans (Cramer’s *V* = 0.03, *p* < 0.05). A smaller proportion of AIAN veterans reported debt (Cramer’s *V* = 0.05, *p* < 0.05) and severe mental illness (Cramer’s *V* = 0.03, *p* < 0.05) compared to non-AIAN veterans. Finally, a greater proportion of AIAN veterans reported previous jail experience (Cramer’s *V* = 0.04, *p* < 0.05) and alcohol abuse (Cramer’s *V* = 0.04, *p* < 0.05). Finally, significantly more AIAN veterans were rural (Cramer’s *V* = 0.11, *p* < 0.05) and frontier (*d* = 0.19, *p* < 0.05) residents compared to non-AIAN veterans.

### Proportion of psychiatric disorders by housing status and AIAN status

To examine our first two aims, Tables 2, 3 display the proportion of psychiatric disorders by housing status and AIAN status. Results indicated distinct patterns for each psychiatric condition. A greater proportion of HUH AIAN veterans experienced alcohol use disorder (30.41% vs. 23.73%) and PTSD (23.82% vs. 17.55%) compared to non-AIAN counterparts. After controlling for AIAN identity, the strongest association was observed between housing status and PTSD (OR 1.95; 95% CI [1.70–2.23]). The second strongest association was found between housing status and alcohol use disorder (OR 1.54; 95% CI [1.34–1.74]). Finally, housing status was also significantly associated with depression (OR 1.27; 95% CI [1.11–1.44]).

**Table 2:** Proportion for psychiatric disorders for housing status and American Indian and Alaska Native (AIAN) status.

Psychiatric disorders		Non-AIAN			AIAN		
		HUH	SH	OR	HUH	SH	OR
		*n* (%)	*n* (%)	(95% CI)	*n* (%)	*n* (%)	(95% CI)
Suicidal ideation	Yes	574 (10.53)	104 (8.01)	0.74^*^ (0.59–0.92)	24 (7.52)	4 (6.90)	0.91^**^ (0.22–2.80)
	No	4,879 (89.47)	1,194 (91.99)		295 (92.48)	54 (93.10)	
Alcohol use disorder	Yes	1,294 (23.73)	415 (31.97)	1.54^*^ (1.32–1.73)	97 (30.41)	28 (48.28)	2.14^*^ (1.16–3.92)
	No	4,159 (76.27)	883 (68.03)		222 (69.59)	30 (51.72)	
Drug use disorder	Yes	1,059 (19.42)	234 (18.03)	0.91^**^ (0.77–1.07)	61 (19.12)	11 (18.97)	0.99^**^ (0.44–2.08)
	No	4,394 (80.58)	1,064 (81.97)		258 (80.88)	47 (81.03)	
Schizophrenia	Yes	209 (3.83)	19 (1.46)	0.37^*^ (22–0.60)	8 (2.51)	2 (3.45)	1.39^**^ (0.14–7.21)
	No	5,244 (96.17)	1,279 (98.54)		311 (97.49)	56 (96.55)	
Psychosis	Yes	140 (2.57)	27 (2.08)	0.81^**^ (0.51–1.23)	8 (1.88)	2 (5.17)	2.85^**^ (0.45–13.74)
	No	5,313 (97.43)	1,271 (97.92)		313 (98.12)	55 (94.83)	
Depression	Yes	1,431 (26.24)	396 (30.51)	1.23^*^ (1.08–1.41)	73 (22.88)	23 (39.66)	2.21^*^ (1.17–4.13)
	No	4,022 (73.76)	902 (69.49)		246 (77.12)	35 (60.34)	
Bipolar disorder	Yes	406 (7.45)	88 (6.78)	0.90^**^ (0.70–1.15)	13 (4.08)	1 (1.72)	0.41^**^ (0.01–2.86)
	No	5,047 (92.55)	1,210 (93.22)		306 (95.92)	57 (98.28)	
Mood disorder	Yes	285 (5.23)	67 (5.16)	0.98^**^ (0.74–1.30)	10 (3.13)	3 (5.17)	1.68^**^ (0.29–6.82)
	No	5,168 (94.77)	1,231 (94.84)		309 (96.87)	55 (94.83)	
Phobia	Yes	35 (0.64)	13 (1.00)	1.57^**^ (0.76–3.04)	1 (0.31)	0 (0.00)	-
	No	5,418 (99.36)	1,285 (99.00)		318 (99.69)	58 (100)	
Generalized anxiety disorder	Yes	288 (5.28)	82 (6.32)	1.21^**^ (0.93–1.56)	5 (1.57)	4 (6.9)	4.65^*^ (0.89–22.24)
	No	5,165 (94.72)	1,216 (93.68)		314 (98.43)	54 (93.10)	
Obsessive-compulsive disorder	Yes	22 (0.4)	3 (0.23)	0.57^**^ (0.11–1.91)	1 (0.32)	0 (0.0)	-
	No	5,431 (99.60)	1,295 (99.77)		318 (99.69)	58 (100)	
Anxiety	Yes	835 (15.31)	218 (16.80)	1.12^**^ (0.94–1.32)	40 (12.54)	40 (17.24)	1.45^**^ (0.61–3.21)
	No	4,618 (84.69)	1,080 (83.20)		279 (87.46)	48 (82.76)	
Posttraumatic stress disorder	Yes	957 (17.55)	379 (29.20)	1.94^*^ (1.68–2.23)	76 (23.82)	23 (39.66)	2.10^*^ (1.11–3.91)
	No	4,496 (82.45)	919 (70.80)		243 (76.18)	35 (60.34)	
Adjustment disorder	Yes	351 (6.44)	83 (6.39)	0.99^**^ (0.77–1.28)	15 (4.70)	2 (3.45)	0.72^**^ (0.08–3.25)
	No	5,102 (93.56)	1,215 (93.61)		304 (95.30)	56 (96.55)	
Other mental health disorders	Yes	413 (7.57)	89 (6.86)	0.90^**^ (0.70–1.14)	13 (4.08)	7 (12.07)	3.21^*^ (1.04–9.18)
	No	5,040 (92.43)	1,209 (93.14)		306 (95.92)	51 (87.93)	

HUH, Homelessness and Unstable Housing. ^*^p < 0.05, ^**^p = n.s.

**Table 3:** Overall odds ratios (ORs) for psychiatric disorders prevalence in veterans stratified by American Indian and Alaska Native (AIAN) status, and AIAN status as moderator for housing status and psychiatric disorders.

Psychiatric disorders	Moderation	
	OR_MH_ (95% CI)	AIAN Identity as *Z* $\left(p_{\mathrm{BD}}\right)$
Suicidal ideation	0.75^*^ (0.60–0.92)	n.s.
Alcohol use disorder	1.54^*^ (1.34–1.74)	n.s.
Drug use disorder	0.92^**^ (0.79–1.07)	n.s.
Schizophrenia	0.40^*^ (0.26–0.63)	n.s.
Psychosis	0.87^**^ (0.59–1.30)	n.s.
Depression	1.27^*^ (1.11–1.44)	n.s.
Bipolar disorder	0.89^**^ (0.70–1.13)	n.s.
Mood disorder	1.01^**^ (0.77–1.31)	n.s.
Phobia	1.53^**^ (0.81–2.90)	n.s.
Generalized anxiety disorder	1.26^**^ (0.98–1.61)	n.s.
Obsessive-compulsive disorder	0.55^**^ (0.17–1.85)	n.s.
Anxiety	1.13^**^ (0.96–1.32)	n.s.
Posttraumatic stress disorder	1.95^*^ (1.70–2.23)	n.s.
Adjustment disorder	0.98^**^ (0.77–1.26)	n.s.
Other mental health disorders	–^***^	< 0.01

^*^p < 0.05, ^**^p = n.s.; OR_MH_, Mantel–Haenszel Odds Ratio with Wald Confidence Intervals; *p_BD_*, Breslow-Day of homogeneity of ORs.

***“The Mantel–Haenszel adjusted measures of association are valid when the measures of association across the different strata are similar (homogenous), that is when the test of homogeneity of the odds (risk) ratios is not statistically significant” (44, p. 10).

### Housing status and service needs

HUH AIAN veterans (46.08%) reported higher psychiatric needs than SH AIAN veterans (34.48%). Regarding medical services, HUH veterans (61.13%) were nearly twice as likely to report needing medical help compared to SH AIAN veterans (32.76%) (OR = 0.31). In contrast, substance abuse treatment needs among AIAN veterans showed a different trend. SH AIAN veterans (59.60%) report higher substance abuse services need compared to HUH veterans (35.42%) (OR = 2.40). Regarding case management and family services, results indicated that case management needs were high across both groups, whereas family service needs were low and similarly reported by both SH and HUH AIAN veterans.

### Housing status and service seeking willingness

Across the services, HUH AIAN veterans were generally more willing to seek services across most categories, especially in substance abuse and family services. Among HUH AIAN veterans, willingness to get services was highest for case management services (98.93%) and lowest for substance abuse treatment services (74.77%). Similarly, among SH AIAN veterans, the willingness to get services was highest for medical and family services (100%) and lowest for psychiatric services (85%).

### Moderation results

To assess whether AIAN status moderates the relationship between housing status and psychiatric conditions, we utilized the Breslow-Day test. Results revealed a significant interaction effect, demonstrating that the association between housing status and other mental health disorders significantly differed between AIAN and non-AIAN individuals (χ^2^*_BD_* (1) = 6.34, *p* < 0.05) (Table 3).

We also checked whether AIAN status moderates the relationship between housing status and service needs as well as willingness to seek care. The Breslow-Day test indicated that the association between housing status and psychiatric service needs (χ^2^*_BD_* (1) = 11.63, *p* < 0.05), willingness to seek psychiatric services (χ^2^*_BD_* (1) = 4.91, *p* < 0.05), and medical treatment service needs (χ^2^*_BD_* (1) = 7.94, *p* < 0.05) differed significantly between AIAN and non-AIAN (see Table 4).

**Table 4:** Service needs and willingness to seek help among veterans and American Indian and Alaska Native (AIAN) identity as moderator.

Service categories		Non-AIAN			AIAN			Interaction	
		HUH (%)	Stably Housed (%)	OR (95% CI)	HUH (%)	Stably Housed (%)	OR (95% CI)	AIAN Status as *Z (p_BD_)*	OR (95% CI)
Psychiatric treatment	NH	3,134 (57.47)	912 (70.26)	1.75^*^ (1.53–2.00)	147 (46.08)	20 (34.48)	0.61^**^ (0.33–1.14)	< 0.01	–^***^
	DNH	2,139 (42.53)	386 (29.74)		172 (53.92)	38 (65.52)			
	WGH	2,519 (86.80)	838 (95.55)	3.27^*^ (2.32–4.71)	124 (89.21)	17 (85.00)	0.69^**^ (0.17–4.08)	< 0.05	–^***^
	NWGH	383 (13.20)	39 (4.45)		15 (10.79)	3 (15.00)			
Medical treatment	NH	3,516 (64.48)	745 (57.40)	0.74^*^ (0.66–0.84)	195 (61.13)	19 (32.76)	0.31^*^ (0.16–0.58)	< 0.01	–^***^
	DNH	1,937 (35.52)	553 (42.60)		124 (38.87)	39 (67.24)			
	WGH	3,370 (97.82)	732 (98.79)	1.81^**^ (0.90–4.13)	184 (95.83)	19 (100)	–	n.s.	1.91^**^ (0.95–3.82)
	NWGH	75 (2.18)	9 (1.21)		8 (4.17)	0 (0.00)			
Substance abuse treatment	NH	1,833 (33.61)	785 (60.48)	3.02^*^ (2.66–3.43)	113 (35.42)	33 (56.90)	2.40^*^ (1.31–4.44)	n.s.	2.99^*^ (2.64–3.37)
	DNH	3,620 (66.39)	513 (39.52)		206 (64.58)	25 (43.10)			
	WGH	1,205 (72.55)	688 (94.38)	6.35^*^ (4.53–9.09)	80 (74.77)	32 (96.97)	10.69^*^ (1.62–454.86)	n.s.	6.47^*^ (4.65–8.98)
	NWGH	456 (27.45)	41 (5.62)		27 (25.23)	1 (3.03)			
Case management	NH	5,049 (92.59)	960 (73.96)	0.22^*^ (0.19–0.27)	291 (91.22)	48 (82.76)	0.46^*^ (0.20–1.14)	n.s.	0.23^*^(0.20–0.27)
	DNH	404 (7.41)	338 (26.04)		28 (8.78)	10 (17.24)			
	WGH	4,841 (98.92)	908 (98.27)	0.62^**^ (0.35–1.17)	278 (98.93)	46 (97.87)	0.50^**^ (0.04–26.62)	n.s.	0.61^**^(0.35–1.06)
	NWGH	53 (1.08)	16 (1.73)		3 (1.07)	1 (2.13)			
Family problems services	NH	805 (14.76)	215 (16.56)	1.15^**^ (0.97–1.35)	57 (17.87)	11 (18.97)	1.08^**^ (0.47–2.27)	n.s.	1.14^**^ (0.97–1.34)
	DNH	4,648 (85.24)	1,083 (83.44)		262 (82.13)	47 (81.03)			
	WGH	469 (72.94)	177 (93.65)	5.46^*^ (2.95–11.1)	44 (88)	10 (100)	-	n.s.	5.62^*^(3.05–10.34)
	NWGH	174 (27.06)	12 (6.35)		6 (12.00)	0 (0.00)			

NH, Need help; DNH, Do not Need Help; WGH, Willing to Get Help; NWGH, Not Willing to Get Help; ^*^p < 0.05, ^**^p = n.s.; OR_MH_, Mantel–Haenszel Odds Ratio with Wald Confidence Intervals; *p_BD_*, Breslow-Day of homogeneity of ORs.

^***^“The Mantel–Haenszel adjusted measures of association are valid when the measures of association across the different strata are similar (homogenous), that is when the test of homogeneity of the odds (risk) ratios is not statistically significant” (44, p. 10).

## Discussion

This study is one of the few to explore mental health conditions, service needs, and service-seeking willingness among AIAN and non-AIAN veterans. Our findings reveal significant differences between AIAN and non-AIAN veterans across several demographic and health characteristics. First, AIAN veterans tend to be younger, have shorter military service periods, are more likely to be employed, report poorer health, and require fewer services than non-AIAN veterans. Second, we found that larger proportions of AIAN veterans are female, married, exposed to combat, served in the Army, and have prior experiences with incarceration or substance abuse, as well as frequent ER visits than non-AIAN veterans. These socioeconomic and military characteristic differences between AIAN and non-AIAN veterans align with national data and highlight the documented health and housing disparities among AIAN veterans (1, 12, 15).

A slightly and significantly higher proportion of AIAN veterans were found to be HUH compared to non-AIAN veterans. These findings are consistent with previous research that emphasizes the greater prevalence of homelessness among AIAN veterans (7, 9). The differences in housing status between the two groups underscore the unique challenges faced by AIAN veterans, which may be influenced by socioeconomic, sociocultural, and historical factors specific to this population.

In our sample, AIAN veterans consistently showed higher prevalences of alcohol use disorder and PTSD across both the HUH and SH subgroups compared to their non-AIAN counterparts. Interestingly, for schizophrenia, psychosis, depression, and other mental health disorders, the prevalence tends to be higher in the AIAN SH group but lower or similar in the AIAN HUH group compared to non-AIAN. Our results are generally consistent with previous findings showing that AIAN veterans are at greater risk for mental health conditions (2, 20–22). However, these prevalent patterns raise important questions that warrant further investigation. One possible explanation for the lower prevalence of certain psychiatric disorders among HUH AIAN veterans could be underdiagnosis rather than a true lower occurrence of these conditions.

Regarding mental health and service needs, we found that HUH AIAN veterans were more likely to require psychiatric treatment services to their SH counterparts. Additionally, the need for medical services was nearly twice as high among HUH veterans (61.13%) compared to SH AIAN veterans (32.76%). In contrast, substance abuse treatment needs among AIAN veterans showed a different trend. SH AIAN veterans report higher substance abuse services need compared to HUH veterans. This study also revealed that a greater proportion of SH AIAN veterans were willing to seek various forms of psychiatric and family services compared to non-AIAN veterans. This could be attributed to the availability of culturally tailored services within the VA system, which may enhance engagement and treatment adherence among AIAN veterans (45). While some studies indicate higher healthcare utilization among AIAN veterans (26), others suggest barriers to healthcare access, particularly for those living in frontier or remote areas (41).

Finally, we found a significant interaction effect among AIAN versus non-AIAN veterans for the association between housing status and other mental health disorders. This finding suggests that the relationship between housing status and psychiatric disorders varies across different psychiatric conditions when AIAN identity is considered. Additionally, our results revealed that the relationship between housing status and psychiatric service needs, willingness to seek psychiatric services, and medical treatment service needs differed significantly between AIAN and non-AIAN veterans, further demonstrating a significant interaction effect. More specifically, among non-AIAN, SH veterans reported more need and willingness for psychiatric services, while among AIANs, the pattern for need was reversed since HUH AIAN veterans reported more need for psychiatric services. These findings may reflect a complex interplay of factors, including systemic barriers such as limited availability of culturally relevant services, social determinants of health like poverty and limited access to transportation, and cultural considerations such as historical trauma and mistrust in the VA healthcare system. The significant interaction between AIAN status and housing status highlights the importance of considering AIAN identity when assessing mental health needs in the context of housing instability. The interaction effect suggests that the mental health impact of housing instability may differ for AIAN veterans, possibly due to socio-cultural factors, including systemic challenges in accessing culturally competent mental health care (28, 42).

### Implications

Our findings have multiple clinical, research, and policy implications. First, our findings highlight particular healthcare needs of AIAN veterans who are HUH, which suggest potential targeted interventions. It is important to remember that, compared to non-AIAN veterans, more proportion of AIAN veterans live in rural or frontier locations—where healthcare resources and services are limited. Our sample was more urban-based so there may be limited generalizability to AIAN veterans living in less populated areas. Nevertheless, geographic isolation remains an important consideration for future research, especially in states like North and South Dakota, Iowa, Minnesota, and Nebraska, where frontier and remote populations are significantly higher than the national average (37). Future research should examine how place-based factors—such as rurality, social isolation, and access to culturally responsive services—interact with housing and mental health outcomes.

In terms of policy implications, our study suggests that addressing the housing and healthcare needs of AIAN veterans requires consideration for both the historical and geographical factors influencing their access to services. Culturally tailored initiatives may improve engagement with healthcare services and address health disparities in this population. Policies should aim to increase the availability of culturally tailored health services and ensure that such services are accessible in rural, frontier, and remote areas. In particular, improving the reach of the VA’s culturally competent care could help alleviate some of the barriers to service utilization among AIAN veterans (45).

Our findings also underscore the critical importance of early identification and continuous monitoring of HUH risk among AIAN veterans—particularly during the transition from military to civilian life. Timely and ongoing assessment of risk and protective factors of HUH such as mental health conditions, unmet healthcare needs, employment status can enable targeted interventions before HUH crisis occur. Embedding standardized homelessness risk screening into routine VA and non-VA healthcare and transition programs may allow healthcare providers to proactively identify at-risk AIAN veterans and connect them with supportive services, housing resources, and behavioral health care at the earliest signs of need. By shifting the focus from reactive responses to preventive strategies, the VA and non-VA healthcare systems can play a vital role in reducing or eliminating veteran homelessness and promoting long-term housing stability.

This study has several limitations that warrant consideration. First, our sample was confined to veterans in VISN 23, which may limit the generalizability of our findings to the broader U.S. veteran population. Future research should examine these relationships in nationally representative samples to better understand the prevalence and impact of housing instability, unmet healthcare needs (HUH), and mental health among AIAN veterans across different regions. Second, the cross-sectional nature of the data limits our ability to establish causal relationships between housing status, psychiatric conditions, and service needs. Third, while administrative data and single-item measures offer practical advantages—such as ease of interpretation and reduced participant burden (43)—they may introduce measurement error and limit the depth of assessment. Fourth, the sample includes only veterans engaged with the VA system who completed a housing instability risk screening. This may introduce selection bias and limit generalizability to the broader veteran population, particularly those who seek care exclusively in non-VA settings or who are not connected to formal healthcare systems. Fifth, AIAN status was determined through self-report using a race/ethnicity item, without verification of tribal enrollment. Moreover, the dataset did not include information on specific tribal affiliations. These limitations restrict our ability to explore tribal-level differences that may reflect unique cultural, historical, and geographical contexts influencing healthcare and housing experiences. Future studies should aim to disaggregate AIAN populations to enable more nuanced, culturally responsive research.

In conclusion, this study highlights that AIAN veterans experience higher rates of HUH, greater geographical isolation, and greater barriers to healthcare access compared to non-AIAN veterans. These challenges contribute to a disproportionate burden of homelessness and mental health issues among AIAN veterans. Culturally tailored interventions that consider these unique factors—such as historical trauma, geographic isolation, and access to culturally relevant services—may be critical to addressing health disparities in this population. Future research should continue to investigate how these factors interact to improve the health and well-being of AIAN veterans, with an emphasis on developing effective, culturally sensitive approaches.

## Data Availability

If there is any question regarding data, please reach out to Emre Umucu via eumucu@utep.edu.
